# Editorial: Model organisms in plant science: *Arabidopsis thaliana*

**DOI:** 10.3389/fpls.2023.1279230

**Published:** 2023-09-11

**Authors:** Ali Ferjani, Hironaka Tsukagoshi, Valya Vassileva

**Affiliations:** ^1^ Department of Biology, Tokyo Gakugei University, Tokyo, Japan; ^2^ Faculty of Agriculture, Meijo University, Nagoya, Japan; ^3^ Department of Molecular Biology and Genetics, Institute of Plant Physiology and Genetics, Bulgarian Academy of Sciences, Sofia, Bulgaria

**Keywords:** model organisms, *Arabidopsis thaliana*, fundamental research, plant development, molecular mechanisms, plant-environment interactions

Studies of model organisms not only identify fundamental biological mechanisms but also provide valuable hints and resources for translational research. In the plant kingdom, *Arabidopsis thaliana*, a Brassicaceae family member, holds particular importance due to its distinct advantages for studying plant development and responses to environmental cues. Despite its limited agricultural value, Arabidopsis remains highly favored for physiological, biochemical, genetic, and molecular investigations because of its compact, well-characterized genome, ease of cultivation and manipulation, short life cycle, and prolific seed production (Koornneef and Meinke, 2010). Extensive research on this model organism has advanced plant science globally, giving rise to innovative concepts and methodologies (Chen et al., 2004; Soltis et al., 2007; Van Norman and Benfey, 2009; Krämer, 2015).

The objective of the current Research Topic was to elucidate the importance of Arabidopsis as a model system by presenting recent studies with this species and emphasizing its pivotal role in the progress of plant research.

This Research Topic comprises diverse research articles, including one review, one mini-review, 10 original research papers, and a corrigendum. Together, these contributions explore fundamental aspects of Arabidopsis cell and developmental biology, employing various approaches, including genetics, metabolomics, and phenotypic analysis.

The mini-review by Zhao and Long summarizes the current understanding of how plants respond to mechanical stimuli and translate mechanical information into three-dimensional structures using Arabidopsis organs as a model. Also, the article raises questions about distinguishing mechanical cues, their involvement in cell specification, the management of heterogeneity and robustness, the establishment of mechanochemical hotspots, organ polarity, and the evolutionary preservation of plant shapes under environmental forces.

The review authored by Tabeta et al. focuses on the intricate control of plant leaf size, arising from the complex interplay between genetic and environmental factors. The authors accentuate the pivotal molecular and cellular processes governing organ-wide regulation and the compensatory mechanisms that modulate the leaf size of Arabidopsis (Ferjani et al., 2008). The review also explores novel mechanisms of metabolic and hormonal crosstalk, providing a new research frontier in leaf size regulation.

Soda et al. investigate the molecular basis of stomatal movements in Arabidopsis using the *rtl2* mutant with constitutively open stomata and reduced levels of the enzyme tryptophan synthase ß subunit 1 (TSB1). The *TSB1* gene is involved in the tryptophan biosynthetic pathway, which is also linked to auxin biosynthesis. The authors demonstrate that tryptophan deficiency, not auxin, is responsible for the open stomatal phenotype in the mutant and that external tryptophan application restores the normal stomatal aperture.

The study by Dukic et al. examines the role of chloroplast magnesium (Mg^2+^) in plant and algae photosynthesis. The authors pinpoint three transporters, MGT10, MGR8, and MGR9, in the inner envelope of Arabidopsis chloroplasts. Each of these transporters displays distinct functions in maintaining chloroplast Mg^2+^ homeostasis and regulating photosynthesis. The research shows the significance of MGR8 and MGT10 as key Mg^2+^ transporters while emphasizing the role of MGR9 in plant adaptation to Mg^2+^ fluctuations. Importantly, the authors identify an MGT10 homolog in *Chlamydomonas reinhardtii*, named MRS4, as essential for photosynthesis and cell growth, suggesting an evolutionarily conserved role for these magnesium transporters across species.

An et al. investigate the growth-promoting effects of the brassinosteroid brassinolide (BL) and the fungicide pyraclostrobin (Pyr) on Arabidopsis. The authors demonstrate that the combined application of BL and Pyr increases leaf biomass and inflorescence growth, attributable to improved photosynthetic performance and increased sugar accumulation. The synergistic effect of co-applying BL and Pyr outperforms the effects of individual BR or Pyr treatments. This strategy could potentially offer an environmentally friendlier alternative to mitigate the ecotoxicological impact of Pyr.

A study conducted by Kim et al. explores the role of the root cortex in nutrient and water transport and its selective storage capabilities. The authors examine cortex development and find that cortical cells do not divide during root apical meristem development; however, auxin could trigger cortical cell division, suggesting the role of this hormone in determining the fate of root cortical cells.

Jiang et al. investigate the role of tonoplast proton pumps in plant gametophyte development. The authors observe that the absence of two types of vacuolar proton pumps or a sole deficiency of V-ATPase leads to abnormal development and misplacement of female gametophytes. The lack of V-ATPase activity results in a slowed division of endosperm nuclei following the fertilization of the central cell. This research also reveals that V-ATPase plays a crucial role in regulating auxin levels in ovules, thereby affecting the spacing between the central cell and egg cell nuclei, and subsequently influencing endosperm development.

Regulating intracellular inorganic pyrophosphate (PPi) levels is vital for proper growth and development in living organisms of all kingdoms. In Arabidopsis, an enzyme called vacuolar membrane-bound H^+^-translocating pyrophosphatase (H^+^-PPase)/FUGU5 is important for controlling PPi balance. The study by Tojo et al. focuses on the regulation of PPi levels by examining the impact of H^+^-PPase/FUGU5 deficiency on Arabidopsis. Through phenotypic and metabolomic analyses, this research demonstrates the essential role of H^+^-PPase/FUGU5 in maintaining PPi homeostasis, particularly during the initial stages of seedling establishment, while revealing the minor contribution of the other soluble PPases.

Gunji et al. explore the effects of elevated PPi levels on plant development by taking advantage of the well-characterized loss-of-function H^+^-PPase mutant *fugu5*. The authors demonstrate that excess PPi inhibits gluconeogenesis, disrupts cell division, and triggers compensatory cell enlargement (CCE) in cotyledonary palisade tissue (Ferjani et al., 2011). Excess PPi reduces the complexity of pavement cells and the patterning and functioning of stomata. By selectively removing PPi from specific tissue types, the authors observe improved overall plant growth and suppressed CCE, although this only partially restores epidermal abnormalities. Together, these findings deepen our understanding of leaf metabolic changes, showing tissue- and cell-autonomous growth inhibition due to excess PPi.

Plant micronutrient boron is crucial for rhamnogalacturonan-II (RG-II) pectin polysaccharide crosslinking in the Golgi apparatus, where AVP2;1, a type II H^+^-PPase, is located. Onuh and Miwa study how mutations in the Golgi-localized H^+^-PPase AVP2;1 impact Arabidopsis growth and cell wall components. By applying forward genetics, they find that *AVP2;1* mutations alleviate the inhibition of root cell division and elongation under boron deficiency with no effect under normal conditions. The authors observe a tendency of a reduction in RG-II specific sugars in mutant cell walls, supporting the role of AVP2;1 in pectin synthesis in the Golgi apparatus. Unlike the vacuolar H^+^-PPase FUGU5/AVP1 essential for PPi homeostasis, this study underscores the potential of Golgi-localized H^+^-PPase AVP2;1 in Golgi acidification and cell wall synthesis.

The article contributed by Sotta et al. examines the mechanisms by which overexposure to boron triggers DNA damage and cell death in the root meristems of Arabidopsis. The authors show that in the *rpt5a* mutant of the 26S proteasome, increased boron leads to reduced root elongation, more DNA damage, and cell death. However, introducing a mutation in the NAC domain-containing transcription factor *NAC103*, which is a proteasome target, alleviates these effects. The *nac103* mutation also reduces the accumulation of superoxide in the root tips under boron stress. These outcomes imply that NAC103 contributes to maintaining a healthy root meristem, even in the absence of the RPT5A proteasome subunit under excessive boron.

Uchida et al. employ a metabolome genome-wide association study (mGWAS) to identify genes influencing primary and secondary metabolite contents in Arabidopsis. By analyzing seed metabolomic data acquired through liquid chromatography-mass spectrometry, the authors identify single nucleotide polymorphisms in genes linked to flavonoid and glucosinolate biosynthesis. These researchers also discover an uncharacterized methyltransferase gene associated with N-methylhistidine content and confirm its importance for N-methylhistidine biosynthesis in Arabidopsis through knockout and overexpression experiments.

In summary, the accepted publications provide a comprehensive overview of the recent findings and novel mechanisms that expand our insights into Arabidopsis and plant biology in general. The articles elucidate vital molecular and cellular events that impact plant structure and function, considerably advancing our understanding of plant-environment interactions. These insights hold great promise for driving innovation in crop yield enhancement and opening new avenues of exploration in the field.

## Author contributions

AF: Writing – review & editing. HT: Writing – review & editing. VV: Writing – original draft, Writing – review & editing.
